# Toward communities as systems: a sequential mixed methods study to understand factors enabling implementation of a skilled birth attendance intervention in Nampula Province, Mozambique

**DOI:** 10.1186/s12978-018-0574-8

**Published:** 2018-08-03

**Authors:** Claire B. Cole, Julio Pacca, Alicia Mehl, Anna Tomasulo, Luc van der Veken, Adalgisa Viola, Valéry Ridde

**Affiliations:** 1 Population Services International, 1120 19th St NW Suite 600, Washington, DC 20036 USA; 2 Pathfinder International, 9 Galen Street, Suite 217, Watertown, MA 02472 USA; 3 Pathfinder International Mozambique, 135 Rua Eca De Queiros, Maputo, Mozambique; 4 IRD (French Institute for Research on Sustainable Development), CEPED (IRD-Université Paris Descartes), Universités Paris Sorbonne Cités, ERL INSERM SAGESUD, Paris, France; 5 University of Montreal Public Health Research Institute (IRSPUM), Montreal, Canada

**Keywords:** Maternal health, Skilled birth attendance, Respectful care, Health system strengthening, Community system strengthening, Context, Implementation, CFIR, Mozambique

## Abstract

**Background:**

Skilled birth attendance, institutional deliveries, and provision of quality, respectful care are key practices to improve maternal and neonatal health outcomes. In Mozambique, the government has prioritized improved service delivery and demand for these practices, alongside “humanization of the birth process.” An intervention implemented in Nampula province beginning in 2009 saw marked improvement in institutional delivery rates. This study uses a sequential explanatory mixed methods case study design to explore the contextual factors that may have contributed to the observed increase in institutional deliveries.

**Methods:**

A descriptive time series analysis was conducted using clinic register data from 2009 to 2014 to assess institutional delivery coverage rates in two primary health care facilities, in two districts of Nampula province. Site selection was based on facilities exhibiting an initial increase in institutional deliveries from 2009 to 2011, similarity of health system attributes, and accessibility for study participation. Using a modified Delphi technique, two expert panels—each composed of ten stakeholders familiar with maternal health implementation at facility, district, provincial, and national levels—were convened to formulate the “story” of the implementation and to identify contextual factors to use in developing semi-structured interview guides. Thirty-four key informant interviews with facility MCH nurses, facility managers, traditional birth attendants, community leaders, and beneficiaries were then conducted and analyzed using the Consolidated Framework for Implementation Research through inductive and deductive coding.

**Results:**

The two sites’ skilled birth attendance coverage of estimated live births reached 80 and 100%, respectively. Eight contextual and human factors were found as dominant themes. Though both sites achieved increases, implementation context differed significantly with compelling examples of both respectful and disrespectful care. In one site, facility and community actors worked together as complementary systems to sustain improved care and institutional deliveries. In the other, community actors sustained implementation and institutional deliveries largely in absence of health system counterparts.

**Conclusion:**

Findings support global health recommendations for combined health system and community interventions for improved MNH outcomes including delivery of respectful care, and further suggest the capacity of communities to act as systems both in partnership to and independent of the formal health system.

**Electronic supplementary material:**

The online version of this article (10.1186/s12978-018-0574-8) contains supplementary material, which is available to authorized users.

## Plain English summary

The majority of the causes of global maternal deaths are preventable. This is particularly important in sub-Saharan Africa, where women face a high risk of death during pregnancy and childbirth. Institutional deliveries and provision of quality, respectful care are recognized as critical practices to reduce preventable maternal deaths. Inclusion of the community in maternal health interventions is also a recommended practice. However, there is limited evidence available about what contributes to successful implementation of these practices. In Mozambique, despite a national effort to improve quality, respectful maternal care, the maternal mortality rate has remained among the highest in sub-Saharan Africa. In this study, we selected two facilities in Nampula, Mozambique that sustained high institutional deliveries from 2009 to 2014. We used sequential mixed methods (tracking service delivery coverage, consulting expert panels, and conducting in-depth interviews) to explore how they achieved this. We found that though both sites used the same intervention, their implementation was different. In one site, implementers from the facility and the community collaborated to ensure women received respectful care during institutional deliveries. In the other site, we found examples of disrespectful care, attributed to the facility staff. Community implementers overcame this challenge, relying on each other and using systematic processes to ensure respectful care in their own implementation. These results are useful in considering the extent of communities’ role in practices to promote institutional deliveries and respectful care. They suggest value in investing in communities’ capacity to work collaboratively with and independent of health system actors to sustain implementation.

## Background

Maternal mortality remains a serious and staggering health challenge in sub-Saharan Africa, where women face a lifetime risk of maternal death of 1 in 38 women [1]. Over half of maternal deaths globally are due to preventable causes (e.g. hemorrhage, sepsis, pre-eclampsia) that can be addressed with skilled birth attendance and quality care [2]. The WHO underscores respectful care as critical to quality care that is acceptable to women [2]. Care provided without respect not only violates women’s human rights, but also negatively affects care-seeking behavior, can cause trauma, and can reduce women’s confidence and self-esteem [3].

While there is a growing body of evidence regarding practices to advance respectful, quality maternal care, any evidence-based intervention will face the unknown “black box” of implementation [4–6]. Evidence to support these interventions’ impacts exists, while evidence about their implementation is lacking [2, 7–9]. Understanding what contributes to evidence-based interventions’ successful implementation amidst complexity can support future improvements toward ending preventable maternal mortality (EPMM).

Mozambique’s Model Maternity Initiative began in 2008 as a program of the Ministry of Health emphasizing high impact practice standards across management, service delivery, and community involvement. The initiative prioritized skilled birth attendance through humanizing the birth process; and respectful care in an effort to decrease maternal mortality [10]. Yet during this initiative’s implementation, the maternal mortality rate remained among the highest in sub-Saharan Africa, stagnating at 408 per 100,000 live births [11]. Roughly half of rural births occurred outside of a health facility in 2011, nationally and in Nampula province [11].

From 2009 to 2015, while the United States Agency for International Development-funded *Strengthening Communities through Integrated Programming* (SCIP) project worked to support the Model Maternity Initiative in 14 districts of Nampula Province, project implementers observed increases in institutional delivery rates. In line with the initiative, SCIP implemented an intervention to support respectful care and inclusion of traditional birth attendants (TBAs) in all of the 15 districts’ participating facilities. The intervention consisted of: 1) Engagement with community leaders to reflect on drivers of poor health outcomes, including maternal deaths, and consider solutions. 2) Revitalization of community leadership councils (CLCs) and creation of health facility co-management committees to enable joint health system and community decision-making. As part of their role, CLC members received training and tools for data collection and analysis. 3) Educational community-led dialogue meetings regarding an array of health issues including safe motherhood practices. Facility staff were supported to train community implementers (TBAs and CLC members) to conduct these discussions in their communities. 4) Maternal child health (MCH) nurse and TBA collaboration to build TBAs’ basic safe delivery, antenatal care (ANC), and postnatal care (PNC) knowledge and referral skills, and to prepare them to track beneficiaries from ANC through institutional delivery and postpartum follow up. 5) TBAs’ accompaniment and non-medical attendance of women for institutional deliveries. For further detail of the intervention, see Table 1.

**Table 1:** Detail of Intervention Components

Intervention Component	Description
Engagement with community leaders for reflection on drivers of poor health, and solutions	SCIP facilitated discussions with community leaders and their community members regarding their self-identified health priorities. Community leaders were then supported to apply the results of these discussions to inform health action plans with their community, including actions to address adverse maternal and child health outcomes, among other priorities.
Revitalization of CLCs and creation of health facility co-management committees	Both CLCs and co-management committees are part of government strategies for community engagement, but their implementation is non-uniform. Revitalization efforts focused on ensuring their creation and operational capacity. After this, SCIP provided direct support to council and committee members to structure their meetings, and basic remunerations for transportation. CLCs are village level committees composed of traditional leaders recognized by their community. CLC members help troubleshoot any issues in the community and set agendas for community health and stability. Related to maternal health, CLCs could, for example, provide leadership in building maternal waiting homes, or organize resources for maintenance of bicycle ambulances. Health facility co-management committees bring together facility staff with CLC members, community volunteers, and TBAs to jointly set agendas for health service delivery in response to community needs. Co-management committees met on a monthly to quarterly basis to review and apply data in health service delivery planning. For example, committees could identify the need for improvement in MCH service delivery quality, or to address low ANC attendance by pregnant women in the community.
Educational community dialogue meetings	Communities were supported to begin dialogue meetings regarding their prioritized health topics. Facility staff received training from SCIP as facilitators, and in turn trained CLC members to facilitate these discussions. Sometimes referred to as “Hot Topics” discussions, content ranged from maternal health to agriculture to hygiene. These sessions ensured community access to information regarding effective practices, and the opportunity to consider how these practices might be applied for community benefit.
MCH nurse and TBA collaboration	SCIP supported monthly mentorship meetings between MCH nurses and TBAs. Meetings were held on the facility grounds, and agendas were set by MCH nurses. The content of the meetings focused on building TBAs’ safe delivery, ANC, and PNC knowledge, and community-facility referral skills. TBAs were also supported to track their beneficiaries from ANC through institutional delivery and postpartum follow-up. Meetings provided an ongoing channel for direct communication between MCH nurses and TBAs. Nurses kept attendance sheets and schedules of these meetings, and received follow-up from SCIP coordinators to support their implementation.
TBA escort and non-medical attendance of pregnant women for institutional delivery	TBAs served as liaison for pregnant beneficiaries, coordinating transport for women to the facility and often attending to their needs along the way. Escort typically included negotiating barriers to access such as distance, poor or limited road access and means of transportation, and flooding. Once at the facility, TBAs were to provide non-medical support in line with the Model Maternity Initiative’s focus on humanization of the birth process. For example, TBAs might coordinate family members to be present at the facility during the birth, prepare meals or attend to the comfort of laboring and postpartum women, or support mothers to initiate breastfeeding. MCH nurses retained responsibility for clinical procedures. Under the Model Maternity Initiative guidelines, TBAs were not supported to provide skilled birth attendance [10].

Recognizing the potential to inform future implementation for EPMM interventions, the research sought to explore: What are the contextual factors that may have contributed to the observed increase in institutional deliveries?

## Methods

### Study setting

Mozambique, located in southeastern Africa, is a country in which the majority of its 26.4 million population relies on the public health system for health services [12, 13]. Having moved from a GDP of 5.02 USD Billion in 2000 to 11.01 in 2016, the country has seen improvement in its economic situation in recent years, though in 2009 more than half of the population lived below the poverty line [14]. Maternal health improvement initiatives have been funded by various donors, necessarily engaging all four levels of care in the Mozambican health system [15, 16]. The first point of access to labor and delivery services for the majority of the country’s 70% rural population is through type 2 health centers [11, 17]. These centers are typically staffed with one MCH nurse and up to three maternity beds. Type 2 facilities address complications and need for comprehensive emergency obstetric care through referral to hospitals, which are typically located in district capitals [17]. Type 2 facilities provide Basic Emergency Obstetric Care, with every 2.3 type 2 facilities serving a catchment population of 500,000 on average [17]. This study looks at maternal health related service delivery in two type 2 facilities in the country’s most populous province: Nampula, located in northern Mozambique [11].

### Study design and conceptual framework

We adopted a sequential explanatory mixed methods case study design in which quantitative methods are followed by qualitative methods to explain the results derived from the quantitative phase [18–20]. To ensure thorough reporting of our approach, we describe it here in line with the Mixed Methods Appraisal Tool (MMAT), created in 2014 to support appropriate planning, execution, and appraisal of mixed methods research [20, 21].

Drawing on the positive case study approach, our design identified primary health facilities (herein referred to as “sites”) where clinic data reflected an increase in institutional deliveries, and then constructed the “story” of this increase [22]. We employed three phases: 1) Quantitative analysis of service delivery data to select two positive cases, 2) Integrated analysis of quantitative service delivery data and qualitative data via expert panels to understand the intervention’s implementation, and 3) In-depth interviews with key informants to understand the contextual factors contributing to the increase in institutional deliveries (Table 2).

**Table 2:** The three phases of data collection and analysis, in chronological order according to their implementation

Site selection	
Identification of positive cases	Analysis of clinic register data of skilled birth attendance across intervention districts to identify two primary health care facilities to represent the observed increase in institutional deliveries, in two districts of Nampula Province for study participation.
Integrated qualitative and quantitative analysis	
Service uptake and coverage analysis	Estimated coverage was calculated using demographic health survey and national census data regarding women of reproductive age (15–19) and population size per district to find the estimated number of live births that could be expected in each facility catchment area.
Expert panels (*n* = 20)	Two expert panels (one per site) were convened, each composed of ten experts familiar with maternal health implementation and service delivery efforts at the facility, district, provincial, and national levels. Findings were used to construct timelines of events during the observation period, and to inform development of semi-structured interview guides and stakeholder mapping for respondent selection in subsequent in-depth interviews.
Qualitative analysis	
Key informant interviews (*n* = 34)	• MCH nurses (2, one from each facility) • Facility managers (2, one from each facility) • TBAs (8, from three communities in each facility catchment area) • CLC members (6, from three communities in each facility catchment area) • Beneficiaries (14, from three communities in each facility catchment area) • Intervention implementation manager (1)

### Quantitative process

#### Data collection

The quantitative phase began with site selection in 2011. We used Health and Monitoring Information System clinic register data, collected and verified by the Ministério da Saúde (MISAU), to retrospectively identify facilities exhibiting an increase in institutional deliveries in the 2 years preceding (2009–2011). Though most Nampula districts had observed some level of increase in institutional deliveries in their facilities, five facilities in five districts had significant increases [23]. From these five facilities, three were excluded due to having insufficient clinic register data during the observation period, and poor or absent roads necessary to participate in the study. Institutional deliveries service delivery data in the two remaining primary care facilities continued to be collected from MISAU data sets for the following 3 years, bringing the total years of service delivery observation to five (2009–2014). The two selected primary level facilities and their respective catchment areas are herein referred to as “Upper Province” and “Lower Province” sites (Table 3).

**Table 3:** Characteristics of selected sites

	Upper Province site	Lower Province site
Staff in maternal health ward	1 MCH nurse, 1 facility manager as needed	1 MCH nurse, 1 facility manager as needed
Geographic characteristics	Inland, road infrastructure includes mix of paved and dirt roads. Barriers to facility include distance, rivers prone to flooding, and limited means of transport within community.	Inland, road infrastructure primarily includes dirt roads. Barriers to facility include distance, rivers prone to flooding, and limited means of transport within community.
Estimated catchment population in 2014 (based on 2007 census)	39,964	43,902

#### Data analysis

Institutional delivery data was initially analyzed at the district level. Data from districts representing the observed increase in institutional deliveries was then further examined to identify specific health facilities that demonstrated the trend. Facilities’ service delivery data was subsequently cleaned and verified. Institutional delivery coverage was estimated by comparing the number of reported deliveries with the total number of projected live births (estimated using census data) in each facility’s catchment area. A time series analysis was then conducted, plotting the number of institutional deliveries and coverage rates over the observation years for the two sites, to provide a performance timeline against which the qualitative “story” could be constructed.

### Qualitative process

In 2009, Damschroder et al. conducted a systematic review of 19 articles, themselves comprising over 500 studies of the factors found to influence the implementation quality of evidence-based interventions [24]. Given our interest in explaining the factors that may have contributed to the observed increase in institutional deliveries in the two sites, we determined the authors’ resulting “Consolidated Framework for Implementation Research” (CFIR) as appropriate to our aim and applied it in the data collection and analysis phases of the study, discussed below.

#### Data collection

To understand the increases in institutional deliveries, we established a timeline of events. We plotted monthly coverage data from clinic registers in charts, for the facility catchment areas as a whole as well as for each community within it. Communities at a distance equal to or closer than 5 km from the facility were excluded to ensure analysis focused on communities with distance as a common minimum barrier to institutional delivery [25]. We then convened two expert panels of stakeholders (*n* = 10 × 2) reflecting all local levels of the health system with knowledge about maternal health in the two districts. The panels provided independent and then collective input on the stakeholders and events influencing implementation during the observation period [26]. From these inputs, preliminary timeline-based cases of implementation in the two sites were constructed, following a modified version of the Delphi technique [27].

##### Sampling

Building on panel findings, we identified respondents for in-depth interviews according to inclusion criteria. Among implementers, these criteria included involvement in selected facilities’ MCH service delivery efforts during the observation period. Among beneficiaries, these criteria included 1) residence in communities exposed to facilities’ MCH service delivery efforts, and 2) having had a pregnancy during the five-year observation period [28]. Representative groups of stakeholders meeting these criteria were identified in three communities in each site (six total), where communities were selected for their sustained increase in institutional deliveries over the observation period. Owing to logistical and resource constraints (time and funds), we were not able to conduct interviews in more communities. Individual interviewees were identified through project staff and community leaders, and subsequently through snowball sampling until saturation of themes was reached [29].

##### Instruments and field work

Expert panel results were also used to identify the 15 CFIR sub-constructs relevant to the cases, to inform design of the semi-structured interview guide tool [20, 21]. The tool was vetted through back translation. Two male researchers fluent in English and Portuguese conducted the interviews. To address language barriers and any discomfort discussing the topic, two female translators participated in interviews. Translator training included review of the study aims and harmonization of terms between English, Portuguese, and the local language, Emakhuwa.

Thirty-four in-depth interviews were conducted. Nurses and facility managers were interviewed at work; TBAs, community leaders, and beneficiaries were interviewed in their communities. Privacy was ensured in public places by creating quiet spaces to speak. Interviews were reviewed daily with the lead author to ensure observational notes were complete and to determine whether saturation had been reached. Field notes were transcribed, in addition to transcription and translation of audio recorded interviews. An additional team of local translators transcribed from the original spoken language directly to written English.

#### Data analysis

The lead author and an additional analyst who did not participate directly in data collection were responsible for applying CFIR domains for deductive coding, for inductively identifying emerging themes, and for developing a codebook and corresponding code-tree with descriptions of all codes and sub-codes [30, 31]. A two-phase interrater reliability exercise was conducted: The two analysts used line-by-line coding to independently code identical transcripts. The analysts compared their coded transcripts, discussed codes assigned, and refined working definitions for each code as a reference point within the codebook. The analysts continued coding additional transcripts until 80% reliability was reached. Line-by-line coding was then performed for remaining interview transcripts using ImpactMapper. Descriptive summaries of each interview were written and analysts conducted weekly coding checks to ensure continued reliability. Using this process, the analysts coded interviews through deductive and inductive coding, to identify codes specific to the CFIR framework as well as codes relevant but not otherwise captured by the CFIR [32]. Table 4 provides detail of the coding process.

**Table 4:** Coding Process

Process	Illustrative Detail
Expert panel findings inform initial codebook	Expert panel findings regarding the timeline of events during the observation period informed both in-depth interview data collection instruments and the initial codebook. These events included community discussions to identify local health priorities for action, mentorship activities between MCH nurses and TBAs, and the use of evaluative monitoring processes by community leaders, among others. Examples of resulting identified CFIR constructs include: Patient Needs & Resources, Networks & Communications, and Formally-Appointed Leaders.
Inter-rater reliability exercise	Analysts coded a diversity of respondent transcripts independently using CFIR constructs, comparing each other’s coding until 80% reliability was reached.
Combined deductive and inductive coding	Analysts used initial CFIR constructs for coding, and identified emergent themes as they evolved in the coding process. For example, though the CFIR provided codes for implementers’ perception of the intervention and motivation to implement (e.g. Knowledge & Beliefs about the intervention), the framework did not address beneficiaries’ motivation for institutional delivery.
Twice-weekly coding debrief discussions for alignment	As transcripts were coded, the analysts developed narrative summaries of each transcript and codes applied. The analysts then jointly debriefed each transcript, using these as a means of continual alignment in coding and identification of emerging themes. This process was carried through until completion of coding.

## Results

**Fig. 1:**
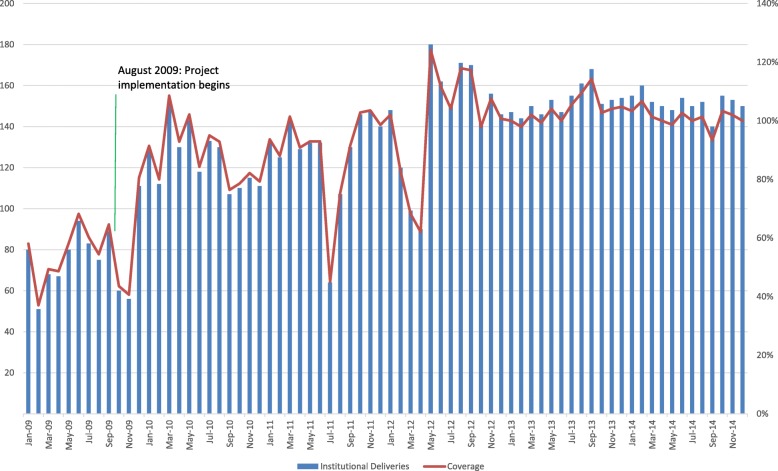
Number and Coverage of Institutional Deliveries- Upper Province, Jan 2009-Dec 2014. Quantitative analysis demonstrates coverage of institutional deliveries in Upper Province increased from 60% to near complete and sustained coverage between 2009 and 2014. Note that coverage rates are estimated using census data. Coverage over 100% may be due to shifts in population size from those reflected in the census, or women traveling from outside the facility catchment area for services

**Fig. 2:**
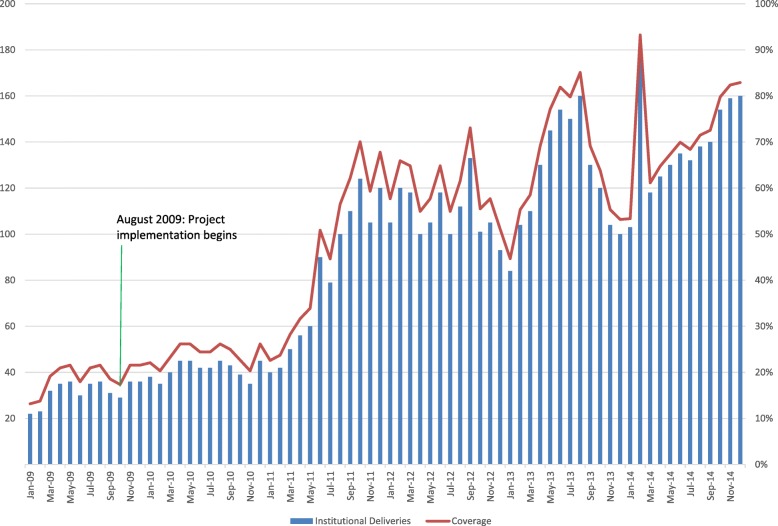
Number and Coverage of Institutional Deliveries- Lower Province, Jan 2009-Dec 2014. Quantitative analysis demonstrates coverage of institutional deliveries in Lower Province increased from just over 10 to 80% between 2009 and 2014

**Fig. 3:**
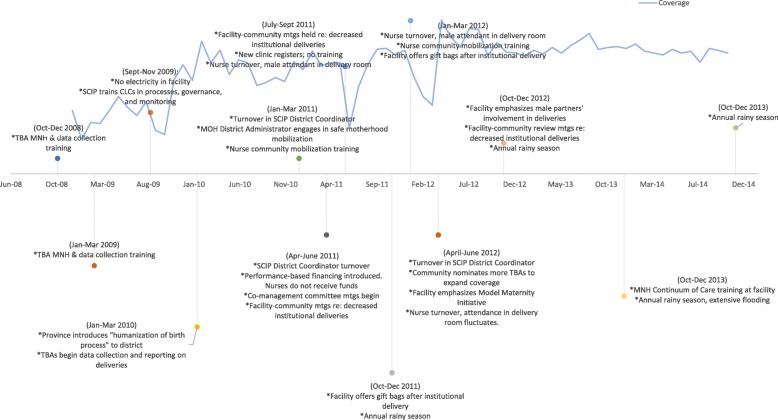
Timeline of Events- Upper Province. Expert panel results construct a story of national-, province-, and community-level events during the observed increase in institutional deliveries, including intervention implementation and frequency of challenges to service delivery such as frequent staffing changes, flooding, and shifts in resource availability

**Fig. 4:**
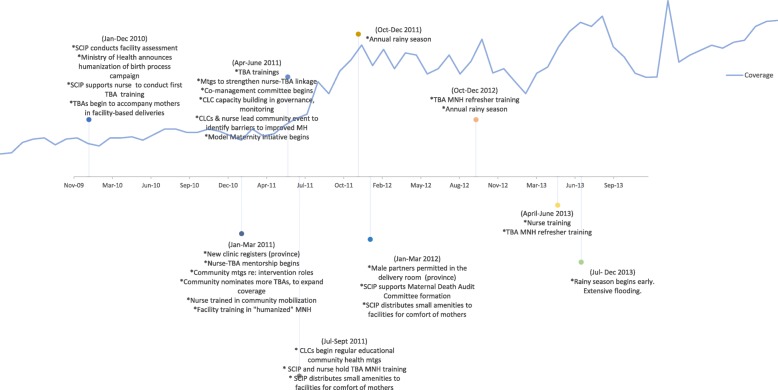
Timeline of Events- Lower Province. Expert panel results construct a story of national-, province-, and community-level events during the observed increase in institutional deliveries. Highlights include a frequency of MNH and TBA refresher trainings, and community efforts to expand the number of trained TBAs in the catchment area

Descriptive time-series analysis showed sustained increases in institutional deliveries. (For number and coverage of institutional deliveries in Upper Province, see Fig. 1. For Lower Province, see Fig. 2.) Institutional deliveries in Upper Province rose from 60% to virtually universal coverage of live births between January 2009 to December 2014, and from 10 to 80% in Lower Province in the same observation period. This high level of coverage, once obtained, was sustained throughout the observation period. The findings from two expert panels, composed of 20 experts in total, subsequently supported creation of a rich timeline of events for each site, reflecting alignment between project implementation and the observed increased and sustained coverage of institutional deliveries. (For the timeline of events in Upper Province, see Fig. 3. For Lower Province, see Fig. 4.)

Analysis of the 34 semi-structured interviews (MCH nurses, facility managers, TBAs, CLC members, beneficiaries, and implementing manager) resulted in eight dominant themes and 11 corresponding sub-themes relevant across both sites. Seven of the dominant themes aligned with constructs encapsulated in the CFIR and one additional dominant theme emerged as a factor influencing implementation that was not otherwise captured by the CFIR constructs: Beneficiary Motivation (Table 5).

**Table 5:** Dominant themes and corresponding definitions

*Intervention Characteristics*	
Adaptability	The degree to which an intervention can be adapted or tailored to local need once in implementation [24].
Patient Needs & Resources	The extent to which the organization understands and prioritizes patient needs and the resources or efforts needed to meet them [24].
*Inner Setting*	
Networks & Communications	The nature and quality of webs of social networks and the nature and quality of formal and informal communications within an organization [24].
Implementation Climate- Compatibility	The fit between an intervention and its implementers’ values, norms, and workflows [24].
*Characteristics of Individuals*	
Knowledge & Beliefs about the Intervention	Individuals’ attitudes toward and value placed in the intervention, as well as familiarity with its underlying facts and principles [24].
Individual Stage of Change	An implementers’ commitment to the intervention, with the end quality of “skilled, enthusiastic, and sustained use [24].”
*Process*	
Engaging Formally Appointed Implementation Leaders	Individuals from within the organization who have been formally appointed with responsibility for implementing an intervention [24].
Beneficiary motivation	Describes the beneficiary’s motivation for engagement in and/or receiving the intervention.

In analysis, the authors found significant overlap between CFIR domains, in particular: Networks & Communications and Knowledge & Beliefs about the Intervention; and Compatibility and Formally-Appointed Leaders. In the following section we have merged overlapping domains to limit redundancy.

### Adaptability

Adaptability was discussed as a factor facilitating implementation by the majority of respondents, particularly in relation to exchange of responsibilities. When needed—most commonly, to attempt to respond to high client volume—TBAs and MCH nurses in both sites interchanged their tasks during deliveries. But actors’ relationship to adaptability and its meaning to their implementation differed between the two sites. In Upper Province, respondents discussed their exchange of roles as a complement to training and an expression of respect and mutual appreciation between TBAs and nurses. Respondents discussed adaptability in the context of mentorship to build TBAs’ skills, and accountability in their division of responsibilities. Nurses also retained the authority over how services should be delivered. As a TBA described:

*“[If] TBAs find the [nurse] with a lot of work, [the nurses] allow…TBAs to assist childbirth and advise…and are there to follow if anything abnormal is happening.”—Upper Province TBA*

In Lower Province, respondents’ relationship to the need for adaptability in implementation varied between stakeholder groups and was not uniformly positive. All respondents described the direct involvement of TBAs in delivery attendance, including the facility nurse who reflected,

“*But now I can even leave [TBAs] alone [to] do the delivery…without any problem. …[If the delivery] is not normal, so together we…help the [laboring client].” –Lower Province Nurse*

However, half of all Lower Province respondents discussed TBAs’ adaptability to attend deliveries alone primarily as a result of facility implementers’ lack of accountability to fulfill their own responsibilities. Both TBAs and beneficiaries recounted births reflecting this:

*“When…the nurse came to observe…she came rather drunk and…said…to [wait]. …After a while [I] gave birth but it was the TBA who assisted.” – Lower Province Beneficiary*

### Patient needs & resources

Nearly all respondents discussed the intervention as responsive to beneficiary needs. First, respondents discussed the intervention’s meeting of clients’ medical needs, particularly regarding unexpected complications:

*“…At the facility…if you have a complicated delivery, can first apply injection and if you have problem giving delivery [of] placenta...[women] are supported. While at the community level that does not happen” --Upper Province TBA*

Beneficiaries’ needs were also described as met by the intervention through introduction of TBAs as escorts to the facility, and to its added emphasis on TBAs’ and nurses’ help to women to find better laboring positions and address their emotional needs:

*“I say some sweet words to her, I moralize her so that she may feel motivated saying that…this child may save her life one day, be a governor, a teacher…I say all this to all women I take to hospital. …in the past I did not…” –Lower Province TBA*

Across the two sites, beneficiaries echoed this perception of a caring, respectful birth experience:

*“After giving birth the TBA looked after me, she washed me and gave bath to my baby and put her on scale to weigh [her], and she took me by the hand and advised me…to try to sleep. After some time when I was still in bed she brought my child…and told me to breastfeed…and…she talked to me.” –Lower Province beneficiary*

In Upper Province, nurses and TBAs shared mutual appreciation of each other’s unique contributions to meeting patients’ needs, both emotionally and medically.

*“When the pregnant woman is in the delivery process [TBAs] help [the beneficiaries] morally. They…are the connect[ing] bond between the pregnant woman and the community.…The pregnant woman feels more protected.”–Facility Manager, Upper Province (01UPF)*

*“…Any emergency…[the nurses] step in”—Upper Province TBA*

In Lower Province respondents pointedly distinguished between implementers when discussing contributions to meeting patients’ needs. Facility staff described themselves and were described by others as meeting patients’ emergency medical needs:

*“I know what to give, and I call for the district, I can call the chief medical officer, I can call the director, I can call the district head of MCH, any [available] line that I can use to communicate, to say that I have a patient to evacuate.” –Lower Province Nurse*

However, across respondent interviews, numerous examples were given in Lower Province in which patients’ needs went poignantly unmet. In all instances, this was discussed as a result of facility staff’s failure to uphold their role as skilled birth attendants, resulting in mistreatment:

*“When I go to call her, the nurse doesn’t come…unless the situation is serious and she needs to come and to transfer the [laboring woman] to another health facility...” –Lower Province TBA*

*“When there is delivery complication and TBA goes to present the case to the nurse, [the nurse] normally answers to go and find out to know if the patient’s family has money. If the family doesn’t have money, the patient won’t be assisted. …the nurse says you have to go to the family to find out if they have 100 meticais” –Lower Province Beneficiary*

### Networks & Communications and Knowledge & Beliefs about the Intervention

The majority of respondents demonstrated deep knowledge of the intervention, and a belief that the intervention’s formally established linkages between community- and facility-based actors contributed to the observed increase in institutional deliveries.

The quality of implementers’ interpersonal relationships as they collaborated came up throughout both sites’ interviews, though not in uniform ways. In Upper Province, collaboration between TBAs and nurses was characterized by mutual appreciation and support:

*“Both of them, both TBA and nurses always worked together. [At] no time…[was] there…someone who said, ‘Let me go to work alone...’ They have been working together. They work well.” –Upper Province Beneficiary.*

By contrast, in Lower Province the quality of relationships between facility- and community-based implementers was less consistent. Though facility-based implementers described a positive working relationship, most community-based implementers portrayed a strained one. Here, a beneficiary echoes this sentiment:

*“…The [TBAs] complain because…the nurses do absolutely nothing especially if the expectant mother comes at night. The nurses just remain in their houses and do not set their feet in the health centre. However, the TBA who came with you…stays by your side” –Lower Province Beneficiary.*

Despite variation in the quality of interpersonal relationships, the intended division of labor was uniformly understood, as were the resulting locusts of control between implementers, which helped inform how and when hand-offs should take place:

*“…The TBAs have the duty of taking the expectant mothers to hospital and hand them to the professional nurses in the maternity. …I mean they work in coordination with health centre personnel…[and, with] the TBAs…the community leaders lead the meetings and they work with us in sensitizing the people”—Upper Province TBA.*

This linkage between facility and community actors was also discussed in detail when respondents described the intervention and their perception of its value. TBA and CLC respondents richly recounted the processes set in place by the intervention and the roles and reinforcing relationships between each other. Community-based implementers seemed to draw pride from their system-like way of generating and sustaining institutional deliveries demand.

Community-based respondents’ pride and belief in their role in the intervention’s effectiveness also appeared to bolster their commitment and ownership of the structured processes they relied on for its implementation, creating a virtuous cycle. For example, rather than deferring to hierarchical processes, in which one might expect community stakeholders to wait for directives, community-based implementers demonstrated ownership of their processes, using them for improvement.

There was a self-starter quality to community implementers’ collaboration, enabled by the organized processes they adopted and maintained. In this way, these themes shared a relationship with implementer’s commitment to implementing the intervention, captured in Individual Stage of Change below.

### Compatibility and Formally-Appointed Leaders

Across sites, respondents demonstrated clarity about the intervention’s formally-appointed leaders. Compatibility between implementers’ roles in the intervention versus their pre-existing social and professional roles facilitated their ability to adopt and implement the intervention, without need to “prove” the appropriateness of their post to their peers. In both sites and across all respondent groups, nurses understood their tasks and were similarly understood and respected as medical authorities by their co-implementers. Similarly, TBAs were regarded and respected for their effective emotional partnership to pregnant and laboring women, an authority that was also uncontested by their co-implementers or beneficiaries. Compatibility brought ease and speed in implementers’ understanding, adoption, and demonstration of mastery in their roles as formally-appointed leaders.

This synergy also facilitated implementers’ ability to overcome emergent challenges to implementation—particularly in the case of CLC members.

*“…Last month we had a situation where…the husband of the pregnant lady, he did not believe she…[needed to deliver in the facility]. [They] came to present the case to the CLC and [we] as members along with the TBAs referred the lady to the health facility.”—Upper Province CLC member*

TBAs and CLC members in particular stewarded and reinforced each other’s leadership roles, leveraging the compatibility of their roles as implementation leaders with their pre-established roles as respected leaders in the community. CLC members upheld TBAs’ leadership over community mobilization and tracking of pregnant beneficiaries from ANC to delivery. TBAs in turn enabled and reinforced CLC members’ authority to review performance data and set agendas for their collective work, and both groups of community-based implementers seemed to have an explicit understanding of the reinforcing value of the contribution of their colleagues’ roles to their own ability to perform effectively.

The authority to collect and use data featured strongly in respondents’ discussion of leadership. Here, the constructs of Engaging Formally-Appointed Leaders and Compatibility overlapped with structured Networks & Communications, as CLC members and TBAs intertwined discussion of their roles as Formally-Appointed Leaders with descriptions of the procedures they used to organize, make sense of, and validate their work—the structures they used as leaders to lead. Through generation and review of their own data, community-based implementers took ownership of their performance and in doing so seemed to draw confidence in the legitimacy of their roles as leaders of the intervention, thereby supporting continued implementation:

*“I know that in this community there is (xxx) number of pregnant women who are about to give birth, or who are still registering their pregnancies, I know everything. I…organize them in this way. …Women who are leaving and women who are pregnant, [so we know] when it is time for [beneficiaries to] give birth.” –Lower Province TBA*

*“TBAs bring [us] the data … how many women are pregnant and how many deliveries they have seen. …Members of the CLC are first to thank the TBAs, [for] the data they provide us, [so we can] know what is the state of health [in] our community...” –Lower Province CLC member*

Though this factor emerged as a facilitator to community-based implementation, it is important to note that in Lower Province, its positive influence did not translate to facility-based implementation. There, where community respondent interviews suggest that nurses and facility managers did not fully uphold their formally-appointed leadership roles, community-based implementers also failed to address this problem as leaders.

### Individual stage of change

Across the two sites, majority of respondents demonstrated ownership of the intervention and their role in its implementation. Facility respondents discussed the importance of sustained collaboration with the community. Community-based implementers similarly described their implementation activities as “permanent” and happening “without fail.” Some of the most powerful statements reflecting implementers’ Stage of Change came as community-based implementers intertwined their reasons for supporting the intervention with stories of sorrow, suffering, and loss due to the previous norm of home births. Respondents recounted examples of family members using pestles to attempt to expedite births by pushing on the fundus of laboring women, protracted and painful labors, lack of hygienic practices, hemorrhage, and death. These experiences seemed to bolster TBA and CLC members’ desire to protect women and families from preventable deaths, in turn fortifying their commitment to delivering change through the intervention.

This commitment to the intervention did not come without cost to implementers. TBAs described long hours and time away from home, the challenges of crossing flooded rivers to escort laboring women to the clinic, pain imposed by the distances traveled, and sleepless nights. Despite these challenges and lack of pay, these stories were interlaced with their expressed intention to sustain implementation.

TBAs also discussed these hardships in terms of stress in their personal lives, as their commitment to ensuring adherence to institutional deliveries challenged their ability to fulfill household and gender roles at home. A TBA poignantly articulated this friction between her commitments:

*“[Women] ask me…‘please escort me [to the facility] I want to give birth,’…I cannot clean my farm, I cannot have my meals at home, there is nothing I can do at home I only leave my husband worried and sometimes angry, but I tell him please…let me go to work. …Even if my husband is in bed and very erected, I…get up and go [to work].” –Lower Province TBA*

TBAs and CLC members’ commitment to sustaining the intervention bore a relationship with their perception of increased recognition, appreciation, and deference from their community as a result of their role in the intervention. This increased social capital may have acted as an informal reward or remuneration for community-based implementers’ efforts, sustaining their resolve and commitment despite lack of pay.

### Beneficiary motivation

Across the two sites, beneficiaries’ views about the intervention and their resulting demand for the services of TBAs, CLC members, and facility staff clearly intermingled with implementers’ own motivation to implement.

Across both sites, motivation based on past negative experience centered on beneficiaries’ previous experiences during births based in the community. Nearly all beneficiaries who discussed their previous births described risk, fear, suffering, and loss of children as motivating factors in adherence to institutional delivery.

*“[I] had a very bitter experience, in the first delivery that…at home, in the community. …It had complications and at the moment [of] the delivery, [I] was unconscious, [I] did not know what was happening and [I] ended up losing the child. …Then [I] started having advices from the [TBAs]. And so when [I had] the second pregnancy, [I knew that] the deliveries are safe when they are done in the maternities…” – Upper Province Beneficiary*

Beneficiaries’ perceived quality of care was a strong motivator as well. Notably, this was also the case in Lower Province, despite respondents’ accounts of misconduct at the hands of facility staff.

*“What motivates us [to come for delivery] even being far away…Is that when we reach the hospital we are well attended [by] the TBAs…The nurses do not appear…they only come to charge 20 meticais...” –Lower Province Beneficiary*

Social momentum arose as a motivating factor in both sites, acting as a sort of peer pressure among community members to continue demand for institutional deliveries.

*“What make [me] decide to give birth in the hospital is the fact that [I] liked to hear that ‘She gave birth in the hospital.’ [I] would not like to hear somebody comment saying that [I] gave birth at home again. [Interviewer:] In your community here, do they talk well about people who give birth in hospital? Yes they do. A person likes it and takes advantage.” —Lower Province Beneficiary*

*“…The tendency…to deliver in the facility, [I don’t] think twice on that. …Other women are [also] aware, they are informed and they are really into the spirit.”—Upper Province Beneficiary*

#### Synthesis of findings

Findings suggest that contextual and human factors had an overlapping and reinforcing relationship in influencing implementation, exhibiting strong “fit” between implementers’ roles and interpersonal relationships as imposed by the intervention, and their pre-existing social and professional roles and relationships. (An additional file, “Reinforcing network of contextual factors influencing implementation” discusses this in greater detail as it relates to the CFIR constructs [see Additional file 1].) Table 6 provides a synthesis of these implementers’ perceptions of the factors influencing their implementation. (An additional file provides greater detail of implementer perspectives aligned to each construct [see Additional file 2]). Implementers’ perspectives in the two sites were not uniform. In Upper Province, facility and community implementers demonstrated shared perspectives on the context surrounding their implementation, and a mutual understanding of each others’ roles in sustaining it. This alignment in perspectives mirrors the alignment that characterized implementation between these groups in Upper Province. By contrast, facility and community implementers’ perspectives of their implementation in Lower Province differed. There, facility staff expressed confidence in meeting patients’ needs, in fulfilling roles and responsibilities, and in their collaborative implementation with community implementers. Community implementers—and particularly TBAs—perceived a strained implementation in which coordination was fractured, and where TBAs bore a disproportional burden of labor, compensating for absent or disrespectful care delivered by facility staff. These discordant understandings of context echo the discordance characterizing implementation between the two groups in Lower Province. However, community implementers’ perspectives converged with each other’s, with TBAs and CLC members’ demonstrating a shared understanding of and collective expectation for their implementation, echoing the alignment that characterized implementation between community implementers.

**Table 6:** Synthesis of implementer groups’ perception of factors influencing implementation: Comparison of Upper and Lower Province Sites

	Upper Province			Lower Province		
Contextual Factors	Community-based Implementers perspective	Facility-based Implementers perspective	Alignment	Community-based Implementers perspective	Facility-based Implementers perspective	Alignment
Adaptability	*Relevant and valued.* TBAs described adaptability between their own and nurse’s roles and responsibilities (e.g. sharing attendance responsibilities during periods of high client volume). TBAs saw this as facilitating implementation.	*Relevant and valued*. Facility staff described adaptability in roles, with TBAs attending to clients during deliveries. Staff valued TBAs’ collaboration to cover client demand, discussing their work as improving client experience.	Y	*Relevant though necessary due to flawed implementation/ imbalance in workload*. TBAs discussed adaptability in roles and responsibilities with facility staff in order to cover client demand. TBAs valued adaptability as a factor enabling sustained implementation, but resented the need for it and attributed this need to perceived poor performance of facility staff.	*Relevant and valued*. Facility staff shared examples of adaptability in roles with TBAs for birth attendance, and perceived this as a positive factor in implementation.	N
Patient Needs & Resources	*Valued and perceived as met sufficiently.* Community-based implementers described the intervention as responsive to patients’ needs, citing examples of patients’ pleasure with the quality and experience of childbirth in the facility and improved outcomes as compared to previous experiences with home birth.	*Valued and perceived as met sufficiently*. Facility-based implementers described the intervention as responsive to patients’ needs, citing examples of patients’ pleasure with the quality and experience of childbirth in the facility and improved client experience as compared to previous experiences when TBAs, male partners, and family members were not involved in facility birth processes.	Y	*Valued but perceived as inconsistently met*. Community-based implementers discussed the intervention as responsive to beneficiaries’ health and emotional needs, and felt this was an important factor. However, TBAs expressed difficulty in ensuring patient needs were met due to examples of disrespectful care and absenteeism on the part of facility staff. These examples were echoed in beneficiary accounts.	*Valued and perceived as met sufficiently*. Facility staff expressed sentiment that patients’ emotional and medical needs were being consistently met by the intervention, attributing this to all implementers.	N
Networks & Communications; Knowledge & Beliefs about the Intervention	*Critical facilitator.* Community-based implementers perceived TBAs, nurses, and health facility personnel as having a strong network and effective communication, which they believed benefited their ability to serve beneficiaries. Respondents valued the support they felt from one and another across community and facility settings.	*Critical facilitator*. Facility-based implementers perceived TBAs, nurses, and health facility personnel as having a strong network that supported effective implementation, sharing examples of how they have learned from community colleagues about how to improve beneficiaries’ comfort and experience in facility-based births.	Y	*Critical but dysfunctional*. TBAs perceived an unequal distribution of work, due to facility staff absences and disrespectful practices toward TBAs and beneficiaries. By contrast, TBAs and CLC respondents perceived themselves to work in a highly functional network that facilitated their implementation of the intervention.	*Critical facilitator*. Facility staff felt that the network between community- and facility-based implementers was important, positive, and a strong facilitator of implementation.	N
Compatibility & Formally Appointed Leaders	*Valued, facilitating implementation.* Community-based implementers described CLCs and TBAs as key influencers and/or gatekeepers in the community, sharing examples of how this aligned with roles in the intervention, and could be leveraged to sustain implementation and community adherence to institutional deliveries.	*Valued, facilitating implementation*. Facility-based implementers recognized their own leadership as medical experts, and also discussed the high level of influence TBAs and CLC members have in setting and enforcing community norms. This was perceived as driving institutional deliveries adherence within the community.	Y	*Valued, facilitating implementation*. Community-based implementers described their ability to leverage their established social stature to organize and track community adherence to institutional deliveries, and similarly saw alignment between facility staff’s intervention roles and their existing medical expertise. This compatibility was perceived as a positive factor in their ability to implement.	*Valued, facilitating implementation*. Facility-based implementers recognized their own leadership as medical experts, and also discussed the high level of influence TBAs and CLC members have in setting and enforcing community norms. This was perceived as driving institutional deliveries adherence within the community.	Y
Individual Stage of Change	*Committed to sustaining the intervention*. Community-based implementers demonstrate their commitment to implementation, even in the face of physical discomfort (injury) and lack of recognition of their efforts. They discuss their work as permanent practice.	*Committed to sustaining the intervention.* Facility-based implementers discuss their new partnership with community implementers as critical and share intention to sustain joint implementation into the future.	Y	*Committed though concerned*. Community-based implementers express personal commitment to their work, even in the face of adversity. However, TBAs and CLC implementers voiced concern that facility implementers do not show the same level of commitment to the intervention, with resulting harmful effects on sustainability.	*Committed to sustaining the intervention*. Facility-based implementers express a positive view of their new partnership with community implementers, discussing it as a permanent part of their work now.	N

## Discussion

Our research supports the relevance of human and contextual factors in the intervention’s successful implementation [24, 33]. In both sites, beneficiaries’ motivations echoed motivations found in the literature. Women’s knowledge of the risks of home birth and perception of better quality care provided through institutional deliveries factored in their decision to deliver at the facility [34–37]. TBAs’ community-based escort to the facility, too, facilitated women’s decision in favor of institutional deliveries [31]. As with beneficiaries, TBAs and CLC members’ belief in the intervention’s benefits to women and newborns supported their motivation to implement, and their use of a structured committee-like system for monitoring and feedback facilitated their implementation [9, 38]. In Upper Province, these TBAs and CLCs played a significant role in the intervention’s successful implementation, working as partners to the health system to provide respectful care throughout the MCH service cascade. Importantly, in Lower Province community actors sustained implementation and delivered respectful care often in absence or in spite of their health system counterparts, which many respondents recounted as having demonstrated neglectful and even unethical behavior toward beneficiaries, despite their training to the contrary [3, 8, 33, 35, 37, 39]. Finally, this study supports a growing body of evidence and guidance pointing to the critical importance of meaningfully engaging community leaders in health care improvement interventions [2, 9, 40–42].

### Relationship of shared context perspectives and coordinated implementation

The role of context, collective action, relational structures, and shared perspectives in empowering women to voice their desires to deliver at the facility and, specific to this study’s focus, facilitating implementation have been found in previous studies [33, 43–45]. This study builds on this evidence base. The contrasting homogeneity versus heterogeneity in implementers’ perspectives on their implementation helped to construct the picture of how these actors worked together as a system [33, 46]. Olivier de Sardan et al. have articulated the roles of congruence (or lack thereof) between patients’ and health workers’ norms on implementation [47]. We additionally find this relevant to health system and community workers.

### A deepening perspective on the role of communities in EPMM

Debpuur et al. have found combined health system and community health coalition-based implementation to be effective [48]. These findings deepen understanding of the extent of communities’ influence in implementation of respectful care and EPMM. Community lay health workers, and particularly traditional healers, have been found to be effective custodians of care, supporting beneficiary satisfaction with services [49]. In this study, community implementers demonstrated high confidence and precise technical understanding of the practices they used to enable their sustained implementation, including techniques for respectful, compassionate care, routine data generation and review, and joint data-based decision-making in pursuit of optimal coverage of beneficiaries. Gimbel et al. have found the significant role that integration of quality improvement into management strengthening can play in unifying health system actors from various levels for sustained implementation [46]. In this case, the intervention’s management strengthening targeted community level actors, and the resulting unification extended beyond the health system, to include the communities’ unified functioning as a system.

Global dialogue has long pointed to the importance of communities’ involvement in global health programming, as lay health workers for MCH care [7], as participants in health care planning and quality improvement [50], and as decision-makers to address their own health needs [51]. However, it has also been recognized that health programs often cite “community empowerment”—e.g. community engagement (passive or active) with information, materials, or skills-building—as a primary objective, rather than working to achieve full community participation [52–55]. In this study, we find community implementers were core contributors to the successful implementation observed in the two sites, enabled by their commitment to the intervention and their capacity to manage their own implementation. Community implementers acted as significant counterparts to the health system in achieving sustained implementation, leveraging their social status to enhance their and their co-implementers’ effectiveness and authority in the intervention, driving demand, influencing community norms, and innovating to ensure service coverage and provision of compassionate, respectful care responsive to women’s emotional and physical needs. The significance of their role in this study echoes the findings of studies in West Africa regarding community leaders’ roles in sustaining performance-based financing schemes [40], and in exercising control over quality and reliability of implementation [56]. The need for this additional influence beyond purely health system-based implementation for quality, respectful care has been well documented [57, 58]. In this case, we find far more than community “involvement” to support the health system’s delivery of quality, respectful care.

Implementers, donors, and decision-makers may use these insights in considering the design of system strengthening programs aimed at sustainable solutions for EPMM and respectful care. Intervention strategies to sustain EPMM interventions may benefit from investing in communities not only to enable their involvement in EPMM, but to enable their full partnership with the health system, as complementary systems for sustained implementation. In this way, when complexity strikes—whether due to logistical or ethical challenges—EPMM interventions will have two capable allies to weather the storm.

### Application of the CFIR

We join a discrete but growing body of studies that have applied the CFIR in low- and middle- income countries (LMIC), and have found the CFIR to offer a useful, evidence-based framework to organize and study complex implementation variations, to gain insight into how and why successful implementation could be achieved in these two sites [46, 59]. Though we were pleased to find the CFIR’s utility to our implementation setting, we also found some challenges to its applicability. First, many complex interventions in LMIC include volunteer-based implementation with actors outside of the formal health system, but the CFIR does not explicitly recognize these implementation settings [24, 30, 60]. Because of this, we were originally unsure of its applicability to our implementation scenario. In the future, it may prove useful to expand the CFIR’s guidance to discuss how the constructs apply specifically to such informal volunteer implementation settings. Second, as discussed above, constructs and sub-constructs often overlapped, shaping how they influenced implementation. Though the CFIR has been recognized for attempting to address the integrated nature of implementation and context [60], we found the framework to lack guidance on this, thereby somewhat lessening our findings’ ability to immediately inform implementers’ real-time decision-making because of the additional interpretation and time required. Related to this, we originally aimed for analysis to be a collective process with implementation managers so as to inform their decision-making [61]. However, by following the recommended process for use of the CFIR—in which it is applied both to inform study design as well as to analyze and code data [31]—our process proved too lengthy and exhaustive for these initial purposes. In the end, we found it necessary to divorce the study from implementation in order to minimize disruption to implementation. As a result, though useful to future implementation, findings could not be used to inform decision-making during the project’s lifecycle. Though recent research by Keith et al. suggest the CFIR may be used for rapid evaluation, we note that these authors were themselves a dedicated research team separate from the implementation team [62]. We suggest the impact of the CFIR may be expanded by design of instruments that can support implementers’ direct and rapid diagnosis of factors influencing evidence-based interventions’ implementation. This may prove particularly useful as implementers take interventions to large-scale and, in so doing, must consider how to adapt and tailor their implementation to exponentially more varied contexts.

### Limitations

Though our study did not aim to measure the impact of the intervention and so did not require it, it was nonetheless not possible to create control sites for the quantitative portion of this study. Consistency and reliability of the clinic register data collected on institutional deliveries and beneficiaries’ community of origin were not uniform in the province. As a result, the authors were required to invest considerable resources to ensure quality data collection in the selected Lower Province and Upper Province sites during the observation period and were not able to invest the same for control sites. Additionally, the authors were able to collect only 8 months of data preceding launch of the SCIP project owing to similar challenges with consistency and reliability of data collection in clinic registers. Our study design has delivered data and findings in which the authors have high confidence. Nonetheless, by nature of our study design, we cannot—and have not aimed to—claim causality between the intervention’s implementation and the observed increases in institutional deliveries, nor generalizability to other settings. Additionally, because of resource constraints similar to those mentioned above, we were unable to include a greater number of communities in key informant interviews. In-depth interview respondents, having been initially identified through facility and project staff, may have introduced bias. To address this, we were careful to ensure that snowball sampling introduced additional respondents in each community and ensured that any new themes triggered subsequent identification of additional respondents until saturation was reached. In analysis, we also used beneficiary respondents’ accounts to verify or validate those of implementers, and vice versa. We also recognize that these sites, as in most of the province, had a previous norm of home births and relatively low health-service-seeking behavior prior to the intervention. As such, our findings regarding respondents’ motivation for institutional deliveries due to perceived quality of care may also be influenced by the relative “novelty” of the facility and may change over time as this novelty reduces and potential exposure to poor quality care at the facility increases. Our observation timeline may not have been sufficient to capture this change. Finally, as this was a study conducted by and for implementers, study limitations must also acknowledge that two of the authors were directly responsible for implementation of the SCIP intervention, and six of the authors have been employed at some time or are employed by the organization that managed the project.

## Conclusions

Study findings provide an explanation of how two primary facilities reached institutional delivery coverage between 80 to 100% of estimated live births in their catchment area. Findings revealed compelling examples of both respectful and disrespectful care, and stark variation in dynamics between facility and community implementing stakeholders as they sustained implementation for EPMM.

This study contributes to the critical dialogue now growing in the field of global MCH regarding EPMM interventions and how to advance provision of quality, respectful care provision. As our field has called for, communities must be involved in EPMM efforts. This study supports a reframing of the extent to which and the intent with which we might approach communities’ engagement. Using the CFIR to systematically examine the implementation factors enabling sustained increase in skilled birth attendance via institutional deliveries—a key element associated with reductions in maternal deaths and disability—we have seen the critical role that respectful, quality care played in fueling implementers’ and beneficiaries’ desire to sustain demand for this EPMM intervention and its implementation [2]. In these two sites, the agents of this respectful care varied—in Upper Province, health system and community actors partnered toward this end. But in Lower Province the failure of health system counterparts to deliver respectful care and the resulting harm caused by this failure allows us to see that community actors acted not in dependence on their health system counterparts but as an autonomous, functional system to sustain respectful care despite their health system counterparts’ failings.

Considering strategies to advance and scale-up EPMM interventions, this study suggests that investment in communities’ capacity as systems—investing in community system strengthening—may be advantageous. In this large-scale intervention, the complexity of real-world implementation introduced variables unforeseen at intervention design. In Lower Province, critical implementation components failed. But in that complexity, the capacity of the community—which operated as a system—was sufficient to compensate. Considering the complex pressures implementers and decision-makers face in securing EPMM interventions’ adoption and use, this may be a wise investment to safe-guard implementation and ultimately ensure that the value of these interventions reach the women and children we intend to serve.

## Additional files

Additional file 1: Reinforcing network of contextual factors influencing implementation. Qualitative analysis detail regarding the reinforcing fit between implementers and the intervention, discussed via the CFIR constructs. (PDF 37 kb)

Additional file 2: Comparison of implementer perspectives of contextual factors influencing implementation in Upper and Lower Provinces. This table provides a visualization of CFIR constructs that emerged as dominant themes in the Upper and Lower Province cases. The table compares perspectives regarding these constructs and their influence on implementation, with illustrative quotes. (XLSX 48 kb)

## Abbreviations

CFIR: Consolidated Framework for Implementation Research
CLC: Community Leadership Council
EPMM: Ending Preventable Maternal Mortality
MCH: Maternal Child Health
TBAs: Traditional birth attendants
