# Supplementary material for: Toward communities as systems: a sequential mixed methods study to understand factors enabling implementation of a skilled birth attendance intervention in Nampula Province, Mozambique
Source: Reprod Health. 2018 Aug 3;15:132. doi: 10.1186/s12978-018-0574-8 (PMC6091088; doi:10.1186/s12978-018-0574-8)
Supplement: Supplementary file 1 — Reinforcing network of contextual factors influencing implementation. Qualitative analysis detail regarding the reinforcing fit between implementers and the intervention, discussed via the CFIR constructs. (PDF 37 kb) [file 12978_2018_574_MOESM1_ESM.pdf]

# Additional File 1: Contextual factors as a reinforcing network influencing implementation

Where findings in Upper Province demonstrated mutual commitment between facility and community implementers to upholding quality, respectful care, in Lower Province findings included alarming examples of disrespectful and dangerously poor-quality care delivered by facility staff. Findings suggest there was a functional relationship between contextual factors influencing this implementation, working in a mutually reinforcing network to support implementers' ability, motivation, and opportunity to collaboratively implement and sustain institutional deliveries and respectful care despite impediments posed.

TBAs, community leaders, and nurses all played roles in the intervention that aligned with their previous professional roles. Prior to the intervention and during, TBAs were advocates and stewards for pregnant women and newborns, community leaders acted as gatekeepers and decision-makers, and nurses provided medical expertise (Compatibility). In line with these roles, implementers' shared perceptions of the intervention as meeting mothers' and infants' needs and reducing maternal deaths (Patient Needs & Resources; Knowledge & Beliefs) contributed to their commitment to its implementation. Implementers' roles in the intervention also re-affirmed their self-identified social roles as care takers of their communities, which in turn contributed to implementers' comfort and confidence in their role in the intervention (Individual Stage of Change), as well as their knowledge of the ways in which their own function in the intervention connected to, was influenced by, and influenced the work of their co-implementers (Networks & Communications). In part because of this strong "fit" between intervention responsibilities and implementers' previous social and intuitive identities, implementers were able to affirm and navigate with ease their work and the work of the peers they relied on, useful in overcoming challenges to implementation.

This "fit" also influenced the processes implementers used to implement. Nurses, community leaders, and TBAs each had designated realms of authority over the intervention's implementation (Formally Appointed Leaders) that built on the pre-existing authority vested in them by their social and professional circles. Implementers leveraged this authority to establish and uphold the structured processes that characterized collaboration in both of the sites (Networks & Communications). Facility staff invoked their authority as medical experts to call community-based implementers for training in safe delivery. TBAs and CLC members used their socially-grounded authority to fulfill their role in convening community educational meetings, influencing community norms, and establishing expectations for safe birth practices. Drawing on this social authority, TBAs tracked their beneficiaries' adherence through the full cascade of care from ANC to institutional delivery, generating data for their own use in monitoring their performance. Reinforcing this structured system, CLC members too leveraged their leadership authority to call regular meetings to evaluate and plan with this data—creating a data generation-to-use cycle within their own communities. And, when instances of non-adherence arose, implementers similarly leveraged their pre-existing authority to course-correct. Community leaders intervened in family's disagreements about whether to seek facility care. When necessary, TBAs pivoted from the limitations formally imposed on their role in birth attendance to their pre-existing social authority as community birth attendants. When a facility nurse was unable or, in the case of Lower Province, unwilling to perform her tasks, TBAs stepped in to ensure patients' needs continued to be met (Adaptability).
